# Supplementary material for: Genome-Wide Characterization of the BTB Gene Family in Poplar and Expression Analysis in Response to Hormones and Biotic/Abiotic Stresses
Source: Int J Mol Sci. 2024 Aug 21;25(16):9048. doi: 10.3390/ijms25169048 (PMC11354360; doi:10.3390/ijms25169048)
Supplement: Supplementary file 1 [file ijms-25-09048-s001.zip › ijms-3147343-supplementary.pdf]

Table S1. The detailed information of *PtrBTB* genes.

Gene Name	Gene ID	Chr	AA	MW	pI	Subcellular Localization
PtrBTB1	Potri.001G086100	Chr01	431	48091.21	4.78	cyto: 6.5, cyto_nucl: 4, mito: 3, chlo: 2, extr: 1, pero: 1
PtrBTB2	Potri.001G096100	Chr01	561	63266.94	5.49	nucl: 9.5, cyto_nucl: 7, cyto: 3.5, plas: 1
PtrBTB3	Potri.001G100900	Chr01	656	73312.38	8.26	chlo: 9, nucl: 5
PtrBTB4	Potri.001G102300	Chr01	478	52455.52	6.12	chlo: 14
PtrBTB5	Potri.001G184200	Chr01	262	28713.47	4.84	cyto: 8, chlo: 3, plas: 1, extr: 1, golg: 1
PtrBTB6	Potri.001G233900	Chr01	503	57778.94	5.44	nucl: 12, chlo: 1, pero: 1
PtrBTB7	Potri.001G255950	Chr01	92	10460.14	9.1	nucl: 8.5, cyto_nucl: 5.5, chlo: 2, cyto: 1.5, mito: 1, golg_plas: 1
PtrBTB8	Potri.001G280100	Chr01	912	103624.85	5.95	E.R.: 4, chlo: 3, nucl: 3, mito: 2, plas: 2
PtrBTB9	Potri.001G295600	Chr01	614	68117	6.82	nucl: 7, chlo: 2, cyto: 2, mito: 2, cysk: 1
PtrBTB10	Potri.001G357100	Chr01	630	70157.57	6.92	nucl: 9, cyto: 5
PtrBTB11	Potri.001G457550	Chr01	1014	114687.42	6.72	chlo: 12, nucl: 1, plas: 1
PtrBTB12	Potri.001G468700	Chr01	330	37324.58	5.65	cyto: 9, nucl: 2, chlo: 1, mito: 1, plas: 1
PtrBTB13	Potri.002G010100	Chr02	801	91483.33	5.73	nucl: 8, cyto: 4, vacu: 1, cysk: 1
PtrBTB14	Potri.002G048300	Chr02	889	100537.77	5.71	nucl: 9, cysk: 2, chlo: 1, cyto: 1, vacu: 1
PtrBTB15	Potri.002G052700	Chr02	704	77845.38	6.5	mito: 6.5, nucl: 4, cyto_mito: 4, chlo: 3
PtrBTB16	Potri.002G056500	Chr02	592	66249.12	6.15	nucl: 5, cyto: 4, chlo: 3, plas: 1, vacu: 1
PtrBTB17	Potri.002G077000	Chr02	273	30974.15	5.08	chlo: 5, nucl: 4, cyto: 3, mito: 1, cysk: 1
PtrBTB18	Potri.002G082800	Chr02	352	40354.16	5.97	mito: 6, chlo: 5, nucl: 2, cyto: 1
PtrBTB19	Potri.002G166700	Chr02	553	62456.74	5.34	nucl: 10, chlo: 2, cysk: 2
PtrBTB20	Potri.002G209700	Chr02	631	70571.36	8.86	chlo: 4, nucl: 4, cyto: 4, mito: 1, cysk: 1
PtrBTB21	Potri.002G242300	Chr02	610	68850.6	6.67	vacu: 5, chlo: 4, nucl: 2, mito: 1, plas: 1, golg: 1
PtrBTB22	Potri.003G129300	Chr03	477	52452.3	5.84	chlo: 12, cyto: 1, vacu: 1
PtrBTB23	Potri.003G131000	Chr03	656	73608.84	8.68	chlo: 8, nucl: 5, cysk: 1
PtrBTB24	Potri.003G135300	Chr03	561	63534.43	5.71	cyto: 5, chlo: 4, nucl: 4, cysk: 1
PtrBTB25	Potri.004G189800	Chr04	627	70523.45	7.55	nucl: 5, cyto: 5, E.R.: 2, plas: 1, vacu: 1
PtrBTB26	Potri.004G194200	Chr04	397	43494.39	6.24	nucl: 5, cyto: 4, chlo: 3, mito: 1, cysk: 1
PtrBTB27	Potri.005G058700	Chr05	554	62616.67	8.73	nucl: 13, chlo: 1
PtrBTB28	Potri.005G075400	Chr05	641	72407.81	6.08	chlo: 5, nucl: 5, mito: 3, E.R.: 1
PtrBTB29	Potri.005G129700	Chr05	518	58784.85	8.51	chlo: 12, nucl: 1, cyto_mito: 1
PtrBTB30	Potri.005G130700	Chr05	667	74690.08	5.31	nucl: 6, chlo: 3, extr: 3, cyto: 2
PtrBTB31	Potri.005G146400	Chr05	626	69594.49	6.82	cyto: 5, nucl: 4, chlo: 2, mito: 2, cysk: 1
PtrBTB32	Potri.005G149400	Chr05	526	59505.52	7.58	nucl: 7, cyto: 4, chlo: 1, vacu: 1, pero: 1
PtrBTB33	Potri.005G178400	Chr05	327	37666.21	5.95	cyto: 11, chlo: 1, nucl: 1, mito: 1
PtrBTB34	Potri.005G183600	Chr05	275	31184.16	5.21	chlo: 4, nucl: 4, mito: 4, cyto: 1, cysk: 1
PtrBTB35	Potri.005G206100	Chr05	586	65869.07	5.79	nucl: 10.5, cyto_nucl: 6.5, cyto: 1.5, chlo: 1, plas: 1
PtrBTB36	Potri.005G210000	Chr05	698	77138.74	6.9	nucl: 4, mito: 4, cyto: 3, chlo: 2, cysk: 1
PtrBTB37	Potri.005G214400	Chr05	894	100983.17	5.61	nucl: 9, cyto: 2, cysk: 2, chlo: 1
PtrBTB38	Potri.005G251300	Chr05	806	92253.14	5.57	cyto: 6, nucl: 5, vacu: 2, cysk: 1
PtrBTB39	Potri.006G003000	Chr06	563	63702.35	8.68	nucl: 4, cyto: 4, golg_plas: 2, plas: 1.5, golg: 1.5, chlo: 1, mito: 1, cysk: 1
PtrBTB40	Potri.006G043400	Chr06	481	52957.36	6.09	chlo: 4, nucl: 2, plas: 2, vacu: 2, E.R.: 2, cyto: 1, mito: 1
PtrBTB41	Potri.006G103400	Chr06	957	108240.52	5.72	chlo: 11, mito: 3
PtrBTB42	Potri.006G148100	Chr06	589	65599.09	5.79	cyto: 5, nucl: 4, chlo: 3, cysk: 2
PtrBTB43	Potri.006G185300	Chr06	446	50114.36	5.32	nucl: 7, mito: 3, cyto: 2, chlo: 1, cysk: 1
PtrBTB44	Potri.006G202900	Chr06	436	49299.43	4.84	cyto: 8, chlo: 3, cysk: 2, nucl: 1
PtrBTB45	Potri.006G264300	Chr06	611	68844.18	8.45	cyto: 5, nucl: 4, mito: 1, plas: 1, vacu: 1, E.R.: 1, golg: 1
PtrBTB46	Potri.007G032800	Chr07	536	60457.43	7.44	cyto: 7, nucl: 4, chlo: 3
PtrBTB47	Potri.007G033900	Chr07	653	73096.8	5.49	nucl: 5, extr: 5, chlo: 3, cyto: 1
PtrBTB48	Potri.007G053200	Chr07	601	67123.51	6.75	nucl: 3, cyto: 3, E.R.: 3, vacu: 2, golg: 2, chlo: 1
PtrBTB49	Potri.007G055100	Chr07	407	46526.97	9.13	chlo: 7, mito: 3, nucl: 1, cyto: 1, extr: 1, vacu: 1
PtrBTB50	Potri.007G093000	Chr07	637	71862.17	6.25	nucl: 5, cyto: 4, chlo: 3, pero: 1, cysk: 1
PtrBTB51	Potri.007G109300	Chr07	546	61431.32	8.51	nucl: 13, pero: 1
PtrBTB52	Potri.007G112600	Chr07	676	76011.25	6.89	nucl: 8, cyto: 3, chlo: 1, vacu: 1, golg: 1
PtrBTB53	Potri.007G118800	Chr07	542	60858.47	9	nucl: 11, chlo: 1, cyto: 1, extr: 1

PtrBTB54	Potri.007G140400	Chr07	407	46060.55	8.82	cyto: 5, E.R.: 4, chlo: 1, nucl: 1, mito: 1, plas: 1, vacu: 1
PtrBTB55	Potri.008G038600	Chr08	627	70095.75	5.22	nucl: 6, cyto: 5, vacu: 2, chlo: 1
PtrBTB56	Potri.008G085500	Chr08	580	64758.16	8.91	cyto: 3, plas: 3, chlo: 2, E.R.: 2, nucl: 1, mito: 1, extr: 1, vacu: 1
PtrBTB57	Potri.008G150100	Chr08	717	79568.04	6.31	pero: 13, cyto: 1
PtrBTB58	Potri.008G186100	Chr08	628	70180.58	9.02	chlo: 4, nucl: 4, cysk: 3, mito: 2, cyto: 1
PtrBTB59	Potri.008G200700	Chr08	409	45428.8	6.5	chlo: 7, mito: 4, cyto: 2, nucl: 1
PtrBTB60	Potri.009G075300	Chr09	912	103562.91	6.08	chlo: 5, nucl: 4, cysk: 3, cyto: 1, mito: 1
PtrBTB61	Potri.009G089500	Chr09	613	67838.86	6.5	cyto: 6, mito: 4, nucl: 3, chlo: 1
PtrBTB62	Potri.009G150500	Chr09	629	70436.5	8.05	nucl: 6, cyto: 4, plas: 2, E.R.: 1, pero: 1
PtrBTB63	Potri.009G156500	Chr09	398	43861.52	5.65	nucl: 5, chlo: 4, cyto: 3, mito: 1, cysk: 1
PtrBTB64	Potri.010G029500	Chr10	410	45551.94	6.6	chlo: 7, mito: 4, cyto: 2, nucl: 1
PtrBTB65	Potri.010G046800	Chr10	628	69963.31	8.91	nucl: 6, cysk: 4, chlo: 3, cyto: 1
PtrBTB66	Potri.010G090900	Chr10	642	70347.37	6.09	cyto: 10, chlo: 1, nucl: 1, pero: 1, cysk: 1
PtrBTB67	Potri.010G223600	Chr10	627	69988.66	5.64	nucl: 8, cyto: 3, chlo: 1, plas: 1, vacu: 1
PtrBTB68	Potri.011G091100	Chr11	629	70013.5	6.16	nucl: 7, cyto: 4, chlo: 1, mito: 1, cysk: 1
PtrBTB69	Potri.012G091400	Chr12	364	41708.31	8.84	nucl: 12, plas: 1, cysk: 1
PtrBTB70	Potri.012G118300	Chr12	480	53943	6.87	nucl: 4, chlo: 3, cyto: 2, plas: 2, mito: 1, pero: 1, E.R._vacu: 1
PtrBTB71	Potri.012G118500	Chr12	587	65419.89	6.31	nucl: 8, chlo: 2, cysk: 2, cyto: 1, golg: 1
PtrBTB72	Potri.013G024400	Chr13	566	64383.18	6.72	nucl: 12, cyto: 1, pero: 1
PtrBTB73	Potri.013G062500	Chr13	303	33543.21	7.04	cyto: 5, chlo: 4, mito: 3, pero: 2
PtrBTB74	Potri.013G083800	Chr13	273	30638.33	5.63	nucl: 11, chlo: 3
PtrBTB75	Potri.013G159000	Chr13	578	64491.19	8.13	chlo: 6, nucl: 4, E.R.: 3, mito: 1
PtrBTB76	Potri.014G093700	Chr14	553	62331.41	5.2	nucl: 11, cysk: 2, chlo: 1
PtrBTB77	Potri.014G133500	Chr14	642	71389.3	8.8	chlo: 6, nucl: 4, cyto: 3, mito: 1
PtrBTB78	Potri.014G163200	Chr14	585	66187.46	5.19	nucl: 7, chlo: 5, plas: 1, cysk: 1
PtrBTB79	Potri.014G164000	Chr14	673	76156.53	8.82	nucl: 10, cyto: 1, plas: 1, cysk: 1, golg: 1
PtrBTB80	Potri.015G087700	Chr15	360	41541.28	8.39	nucl: 13, plas: 1
PtrBTB81	Potri.015G117200	Chr15	587	65389.75	6.07	nucl: 7, cyto: 3, plas: 2, chlo: 1, cysk: 1
PtrBTB82	Potri.016G003700	Chr16	595	67342.3	8.33	cyto: 4, chlo: 3, nucl: 3, plas: 1.5, golg_plas: 1.5, mito: 1, cysk: 1
PtrBTB83	Potri.016G040500	Chr16	443	48839.97	6.37	chlo: 4, nucl: 2, plas: 2, vacu: 2, E.R.: 2, cyto: 1, mito: 1
PtrBTB84	Potri.016G090400	Chr16	623	69636.8	5.12	cyto: 7, nucl: 6, cysk: 1
PtrBTB85	Potri.016G112900	Chr16	462	50144.83	5.23	cysk: 10, nucl: 2, cyto: 2
PtrBTB86	Potri.016G123800	Chr16	961	108480.68	5.72	chlo: 4, nucl: 4, mito: 4, cyto: 1, cysk: 1
PtrBTB87	Potri.016G139900	Chr16	607	67864.92	6.08	chlo: 7.5, chlo_mito: 5.5, nucl: 4, mito: 2.5
PtrBTB88	Potri.017G009700	Chr17	407	46425.23	8.85	nucl: 8, cyto: 2, cysk: 2, chlo: 1, extr: 1
PtrBTB89	Potri.017G041600	Chr17	542	60713.42	9	nucl: 11.5, cyto_nucl: 6.5, chlo: 1, extr: 1
PtrBTB90	Potri.017G048200	Chr17	672	75503.84	6.96	cyto: 7, nucl: 5, chlo: 1, vacu: 1
PtrBTB91	Potri.018G018600	Chr18	612	69014.35	8.86	cyto: 5, nucl: 3, vacu: 3, E.R.: 2, chlo: 1
PtrBTB92	Potri.018G107600	Chr18	446	49974.22	5.57	nucl: 8, mito: 3, cyto: 2, chlo: 1
PtrBTB93	Potri.019G038400	Chr19	301	33369.8	6.08	cyto: 6, chlo: 5, mito: 3
PtrBTB94	Potri.019G039500	Chr19	426	47137.02	5.14	chlo: 9, nucl: 2, cyto: 1, extr: 1, vacu: 1
PtrBTB95	Potri.019G131600	Chr19	575	64136.78	8.27	chlo: 5, nucl: 3.5, E.R.: 3, cysk_nucl: 2.5, mito: 1, plas: 1

Table S2. The *Ka/Ks* ratios of duplication for *PtrBTB* genes.

Gene Pairs		Ka	Ks	Ka/Ks
PtrBTB2	PtrBTB19	0.200578061	2.034319932	0.098597108
PtrBTB2	PtrBTB24	0.058480269	0.218306408	0.267881595
PtrBTB3	PtrBTB23	0.042688202	0.285424536	0.14956038
PtrBTB4	PtrBTB22	0.058821384	0.336693508	0.174703055
PtrBTB9	PtrBTB31	0.236535137	2.433828133	0.097186459
PtrBTB8	PtrBTB60	0.041892306	0.221682506	0.188974342
PtrBTB9	PtrBTB61	0.051433092	0.256823797	0.200266067
PtrBTB10	PtrBTB68	0.045150463	0.209857917	0.215147771
PtrBTB2	PtrBTB76	0.207761623	2.094175048	0.099209291
PtrBTB19	PtrBTB24	0.194388479	1.878995044	0.103453429
PtrBTB13	PtrBTB38	0.044904624	0.222118522	0.202165149
PtrBTB14	PtrBTB37	0.029872031	0.267304024	0.111753016
PtrBTB15	PtrBTB36	0.046668087	0.267183017	0.174667114
PtrBTB16	PtrBTB35	0.095843473	0.239281289	0.400547297
PtrBTB17	PtrBTB34	0.043007996	0.266541578	0.161355673
PtrBTB18	PtrBTB33	0.050884209	0.182874214	0.278247043
PtrBTB15	PtrBTB57	0.164206282	1.914905492	0.085751638
PtrBTB15	PtrBTB66	0.171240814	1.445895342	0.118432371
PtrBTB19	PtrBTB76	0.018242803	0.278264637	0.065559185
PtrBTB20	PtrBTB77	0.026577756	0.224714455	0.11827346
PtrBTB24	PtrBTB76	0.197176875	2.22168303	0.088751128
PtrBTB25	PtrBTB62	0.04065557	0.198059347	0.205269634
PtrBTB26	PtrBTB63	0.034365756	0.256606492	0.133923954
PtrBTB29	PtrBTB46	0.047405739	0.238949802	0.19839204
PtrBTB30	PtrBTB47	0.046328364	0.322620056	0.143600385
PtrBTB27	PtrBTB51	0.063562038	0.316370762	0.200909963
PtrBTB28	PtrBTB50	0.085033515	0.224945947	0.378017544
PtrBTB36	PtrBTB57	0.159935928	1.921864933	0.08321913
PtrBTB36	PtrBTB66	0.170123534	1.608500495	0.105765298
PtrBTB41	PtrBTB86	0.047805098	0.270121789	0.17697609
PtrBTB40	PtrBTB83	0.027255081	0.205392275	0.132697692
PtrBTB39	PtrBTB82	0.076999398	0.295583558	0.260499598
PtrBTB43	PtrBTB92	0.0188811	0.247067662	0.076420768
PtrBTB45	PtrBTB91	0.048527561	0.238952493	0.203084558
PtrBTB52	PtrBTB90	0.041782539	0.264776127	0.15780327
PtrBTB53	PtrBTB89	0.055443955	0.188485821	0.294154515
PtrBTB54	PtrBTB88	0.062628687	0.2692898	0.232569843
PtrBTB59	PtrBTB64	0.031589349	0.252181486	0.125264346
PtrBTB55	PtrBTB67	0.070713045	0.211855047	0.333780317
PtrBTB57	PtrBTB66	0.030555919	0.225800342	0.135322731
PtrBTB58	PtrBTB65	0.041311177	0.210676789	0.196087939
PtrBTB55	PtrBTB84	0.25153271	1.43895057	0.174802884
PtrBTB67	PtrBTB84	0.248739883	1.420492569	0.175108191
PtrBTB69	PtrBTB80	0.06723772	0.345577207	0.194566419
PtrBTB75	PtrBTB87	0.613713204	3.412877088	0.179822826
PtrBTB75	PtrBTB95	0.060459259	0.235630266	0.256585287
PtrBTB73	PtrBTB93	0.052083295	0.13858392	0.375824952
PtrBTB87	PtrBTB95	0.595704696	0	0

Table S3. Primers used in this study.

Gene name	Sequence (5′-3′)
PtrBTB3-F	GGTTGGAGCAGGCGAAGT
PtrBTB3-R	ACGCCTCGCTTTCTCGTC
PtrBTB4-F	CGCACGGTTCCTTGCATG
PtrBTB4-R	ATCAGTGGCCCCAACAGC
PtrBTB13-F	CCGGGCCACAAGCTCTAC
PtrBTB13-R	ATTGTGCGGGTTCGAGCC
PtrBTB16-F	GCCCATGATGTTTGCCGC
PtrBTB16-R	AAGTTCAGGAGACGCCGC
PtrBTB23-F	GTCCACAACCCGACCTGG
PtrBTB23-R	TGGTGGTGGCCATTTGCT
PtrBTB38-F	GATGGGAGGTCGTCGCTG
PtrBTB38-R	TGTTGCTTTGCCCCAGCT
PtrBTB45-F	TTCCTGGTGGGCCAAAGG
PtrBTB45-R	CTCAAGGTACTCGGCCGC
PtrBTB47-F	GAGGGTCCGCGTGGTAAC
PtrBTB47-R	TGCTGCTGCCTTCCCTTC
PtrBTB49-F	GAGCTCCAGTGCCACCTC
PtrBTB49-R	CCTGGTAGCTGTGCGGAC
PtrBTB80-F	TCTCCGGCGACCTCATGA
PtrBTB80-R	GCGGAGGCCATTGGAAGT
PtrBTB95-F	GCAGGTGCACCAAGTCCT
PtrBTB95-R	CGGAGAAGGCAGCAGAGG
Actin-F	ACAGTTCAACCACACTAATGGGCTC
Actin-R	TTTGGCGCACTGAACACTGTAACCAC

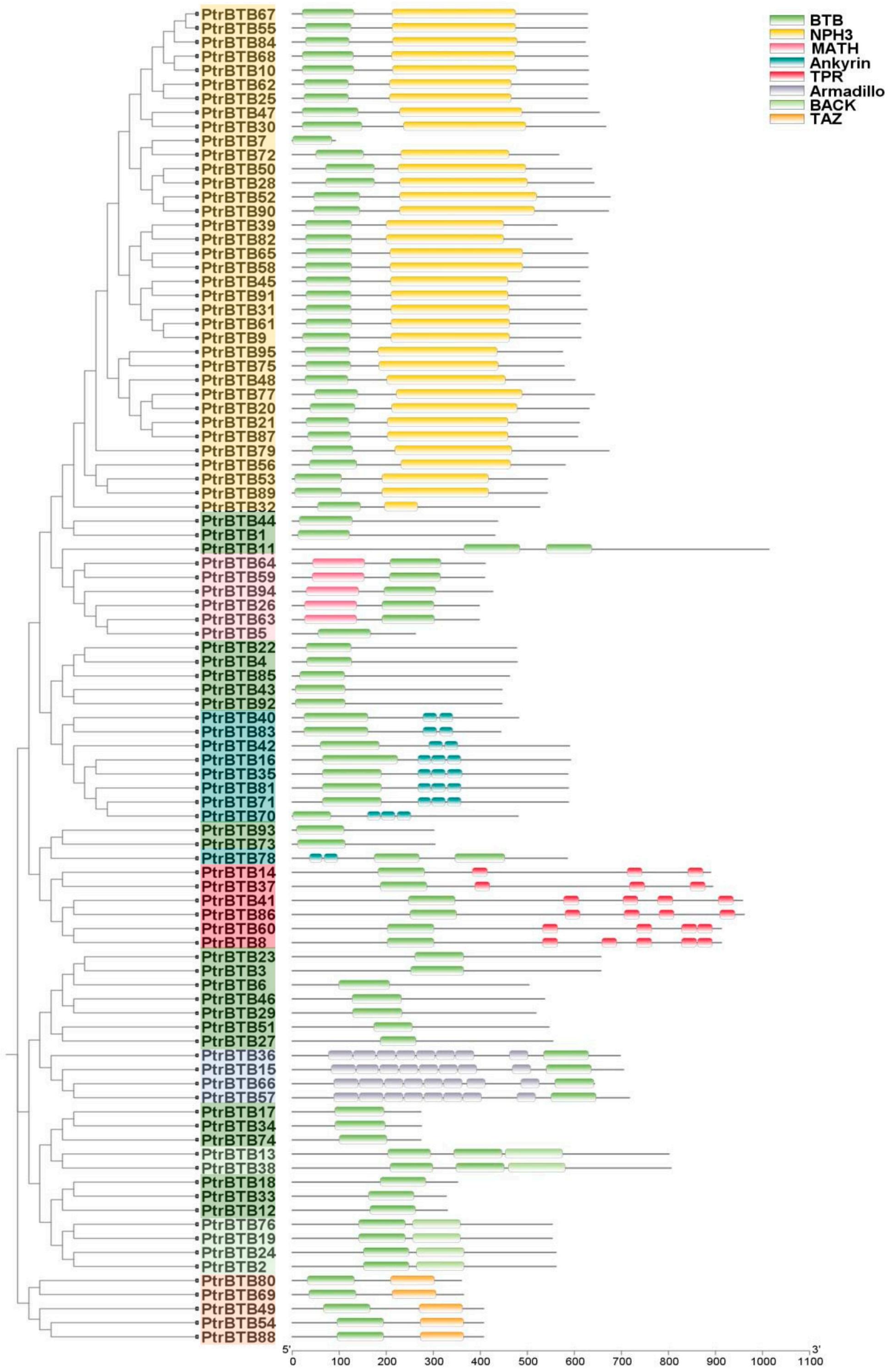

Figure S1. Domains of the PtrBTB proteins. Scale bar represents 100 amino acids. Domains were identified with SMART (http://smart.embl-heidelberg.de/). Different colors represent different domains.

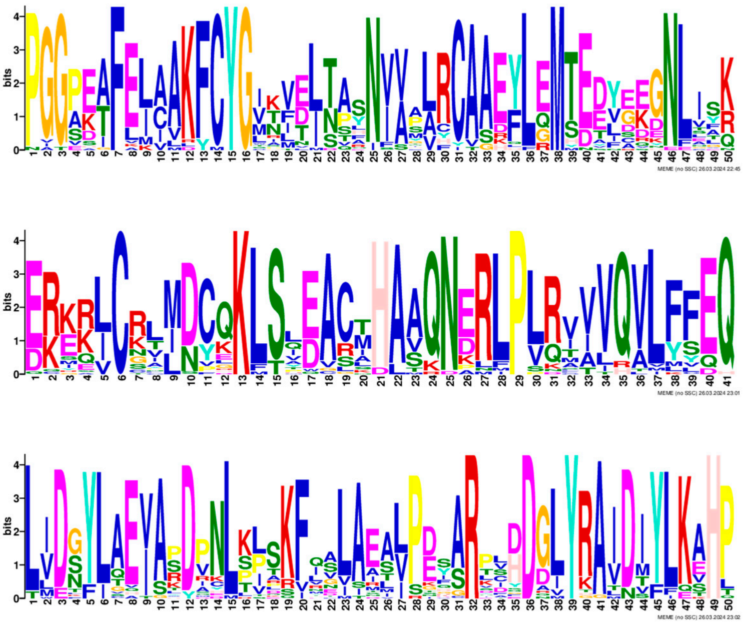

Figure S2. Visualization of multiple sequence alignment of the PtrBTB family DNA binding domains. The total height of the letter piles at each position indicates the conservation of the sequence at that position (measured in bits). The height of a single letter in the letter piles represents the relative frequency of the corresponding amino acid at that position.

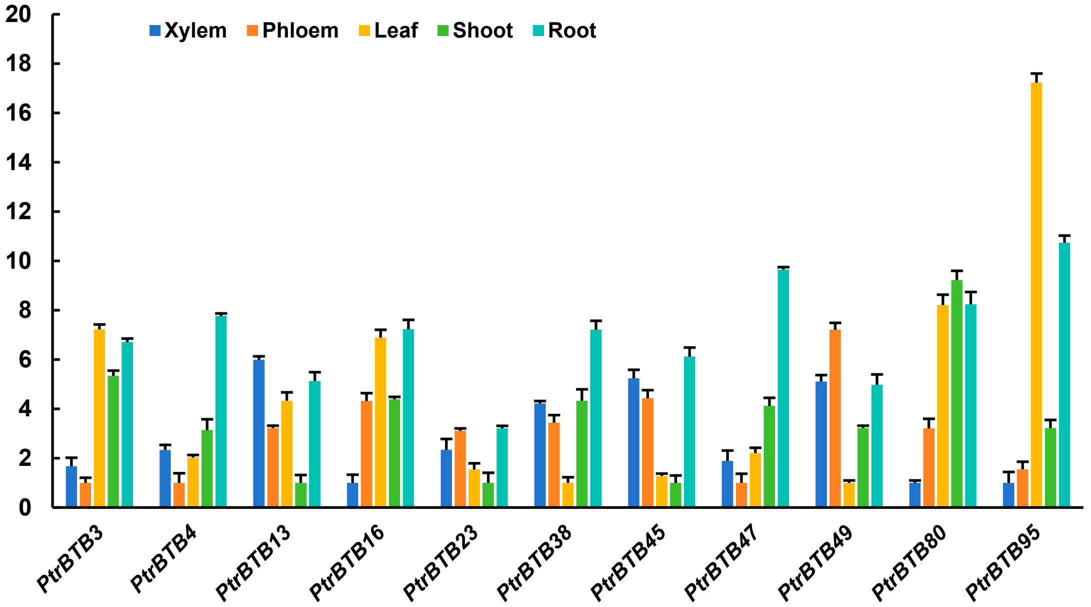

Figure S3. RT-qPCR analysis of the *PtrBTB* genes in different tissues (xylem, phloem, leaf, shoot and root) of 3-month-old poplar 84K. Data are presented as means ± SD of three technical replicates.

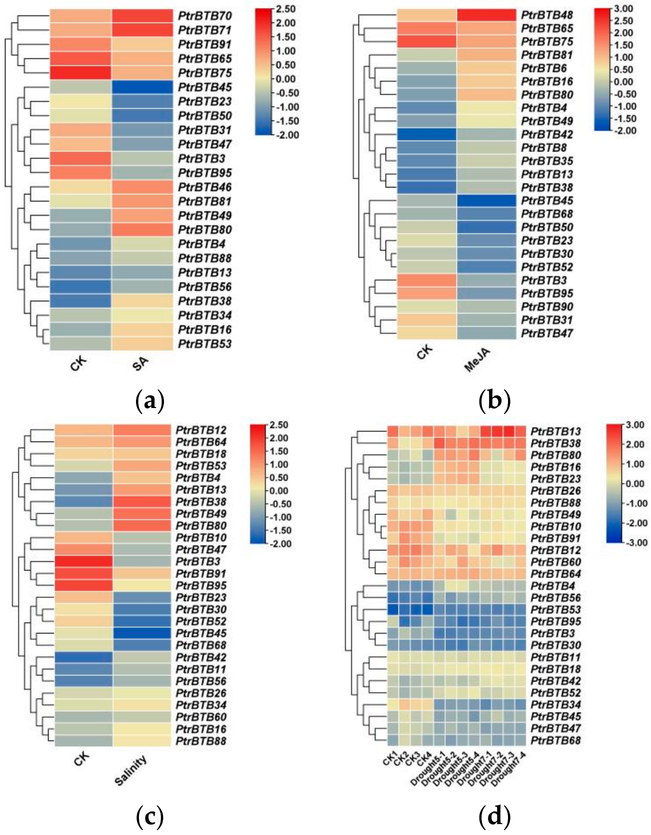

Figure S4. Heatmap of *BTB* gene expression with different treatments in *P. trichocarpa*. (**a**) SA treatment, (**b**) JA treatment, (**c**) NaCl treatment, (**d**) Drought treatment.
